# Proteomic analysis of the faba bean-wheat intercropping system in controlling the occurrence of faba bean fusarium wilt due to stress caused by *Fusarium oxysporum* f. sp. *fabae* and benzoic acid

**DOI:** 10.1186/s12870-023-04481-8

**Published:** 2023-10-06

**Authors:** Bijie Hu, Yiran Zheng, Jiaxing Lv, Jing Zhang, Yan Dong

**Affiliations:** College of Resources and Environment, Yunnan Agricultural University, Kunming, China

**Keywords:** Faba bean, *Fusarium oxysporum f. sp. fabae*, Benzoic acid, Proteomics, Intercropping, Autotoxic substances

## Abstract

**Background:**

In faba bean, continuous cropping severely affects plant growth and increases the incidence of fusarium wilt due to the accumulation of pathogens and autotoxic substances. The intercropping of faba bean and wheat is commonly used to alleviate the occurrence of fusarium wilt in the faba bean.

**Objective:**

To investigate the role of *Fusarium oxysporum f. sp. Fabae*(*FOF*) and benzoic acid in the occurrence of faba bean fusarium wilt and unravel the potential mechanism of intercropping in alleviating its occurrence.

**Methods:**

Hydroponic experiment was carried out using monocropping faba bean (M) and intercropping faba bean and wheat (I) patterns under *FOF* alone stress (M + F, I + F), *FOF* and benzoic acid double stress (M + F + B, I + F + B). The growth of faba bean seedlings under *FOF* and benzoic acid dual stresses were analyzed as well as the protein expression profile of monocropping and intercropping faba bean roots.

**Result:**

Under *FOF* stress, the growth of faba bean seedlings was inhibited, and the inhibitory effect was enhanced under the dual stress of *FOF* and benzoic acid. However, faba bean-wheat intercropping alleviated the inhibitory effect of *FOF* and benzoic acid on faba bean growth. In faba bean, the up-regulated protein was involved in different functions, such as redox, hydrogen peroxide decomposition, and metabolic processes under *FOF* stress (M + F, I + F) compared to the control. Compared with *FOF* stress (M + F, I + F), under the dual stress of *FOF* and benzoic acid (M + F + B, I + F + B), the up-regulated protein in faba bean were involved in intracellular redox balance, defense, and maintenance of cell integrity. Compared with monocropping (M, M + F, M + F + B), the up-regulated protein function of intercropping(I, I + F, I + F + B) was mainly involved in the biosynthesis of secondary metabolites, redox balance, biological carbon fixation of photosynthesis, and so on. KEGG enrichment analysis results showed that intercropping increased ethylene and jasmonic acid synthesis and other related pathways to improve resistance against fusarium wilt in the faba bean.

**Conclusion:**

The growth of faba bean was inhibited under *FOF* stress and the inhibitory effect was enhanced under the dual stress of *FOF* and benzoic acid, which promoted the occurrence of faba bean fusarium wilt. This might be due to the down-regulation of energy and cytoplasmic matrix proteins under *FOF* and benzoic acid stress. The faba bean wheat intercropping alleviated the inhibition of *FOF* and benzoic acid stress by up-regulating the biosynthesis of secondary metabolites, redox homeostasis, photosynthetic carbon fixation, and other related proteins. Besides, it also promoted the biosynthesis of ethylene, and jasmonic acid, improved the resistance of faba bean plants, and alleviated the occurrence of faba bean fusarium wilt. This provides a theoretical basis for the determination of jasmonic acid and ethylene content.

## Introduction

Continuous cropping is largely practiced in the traditional intensive agriculture system. It leads to continuous obstacles in the cropping system, resulting in crop growth inhibition and yield reduction [1]. One of the manifestations of a continuous cropping system is the high incidence of soil-borne diseases. Numerous studies have shown that a high incidence of the soil-borne disease is highly correlated to allelopathy [2]. Autotoxicity is a specific type of allelopathy in which plants release autotoxic substances into the environment through volatile leaching, root exudates, and stubble decay, which adversely affects the growth of the plant as well as the adjacent plants [3]. Phenolic acids are highly active autotoxic substances that are well-studied [4]. Zhou et al. [5] have shown that the growth of cucumber seedlings was significantly inhibited under vanillic acid acid stress. Bao et al. [6] have shown that the 6 phenolic acids secreted by the root system of Panax notoginseng significantly aggravated the soil-borne diseases. Chlorogenic acid secreted by the tomato in the rhizosphere significantly inhibited the growth of its seedlings, resulting in a high incidence of Rhizoctonia root rot [7]. This suggests that phenolic acids secreted by plants enter the soil and inhibit plant seedling growth and root development, leading to a high incidence of soil-borne diseases. Previous studies were mainly focused on the effects of autotoxic substances that were secreted under stress on the occurrence of crop diseases. However, only a few studies have investigated their underlying pathogenic mechanism from the perspective of dual stress of autotoxic substances and pathogenic bacteria.

Previous studies have shown that intercropping is an effective measure to control soil-borne diseases [8, 9]. As shown in the previous study, intercropping of onion, garlic, and cotton remarkably increased the levels of various growth indicators, such as the above-ground dry weight of cotton seedlings, and reduced the incidence of cotton verticillium wilt [10]. Intercropping of maize and capsicum effectively controlled Phytophthora infection in capsicum [11]. In the intercropping system of marigold and tobacco, the root interaction alleviated the occurrence of tobacco wilt by improving growth indicators in tobacco plants [12]. In intercropping of tomato with tiller onion and tall fescue increased plant height and so on, and alleviated the occurrence of tomato diseases [13, 14].This indicates that intercropping reduced the incidence of soil-borne diseases by increasing plant growth and various growth indicators. Previous studies were mainly focused on the effect of intercropping on disease resistance of cash crops; however, only fewer studies have explored food crops, especially in the wheat and the faba bean cropping systems.

Faba bean(*Vicia faba* L.) is a vital legume food crop that is widely grown worldwide as it is rich in carbohydrate and protein content and is one of the most important legume crops in southwest China. However, long-term single planting of faba beans on the same land will result in a high incidence of faba bean fusarium wilt and reduce the yield and quality of faba beans [15, 16]. Faba bean fusarium wilt is a devastating soil-borne disease, which is caused by *Fusarium oxysporum f. sp. fabae*(*FOF*) [17]. Previous studies have shown that intercropping of the faba bean and wheat effectively alleviated the allelopathic autotoxicity of the faba bean, hindering the occurrence of the faba bean fusarium wilt [18]. However, these studies were primarily focused on physiological and biochemical resistance, tissue structural resistance, and other aspects, but did not explore the altered proteome in faba bean fusarium wilt.

Proteomic analysis is commonly used to investigate the altered protein expression profiles of diseased plants [19, 20]. It has become a research hotspot for unraveling the underlying pathogenic mechanism of host plants by analyzing the altered proteome of infected plants. Previous studies have shown that the content of benzoic acid in the rhizosphere soil of mono-cropped faba beans was relatively high [21, 22]. Thus, we hypothesized that benzoic acid is a crucial autotoxic substance that promotes the occurrence of faba bean wilt. In this study, we conducted a hydroponic pot experiment, under the condition of exogenous addition of benzoic acid and inoculation of *Fusarium oxysporum f. sp. fabae* and studied the differences between mono-cropped and intercropped faba bean seedling growth and root protein expression profile. This study aimed to elucidate the pathogenic mechanism of benzoic acid and the molecular mechanism of wheat and faba bean intercropping using proteomics analysis to improve the resistance of faba bean to fusarium wilt. This study could unravel the role of benzoic acid in promoting the development of faba bean wilt and the potential mechanism of intercropping in reducing the autotoxic effect of benzoic acid.

## Results and analysis

### Effect of intercropping under *FOF* and benzoic acid stress on the growth of faba bean seedlings

**Fig. 1:**
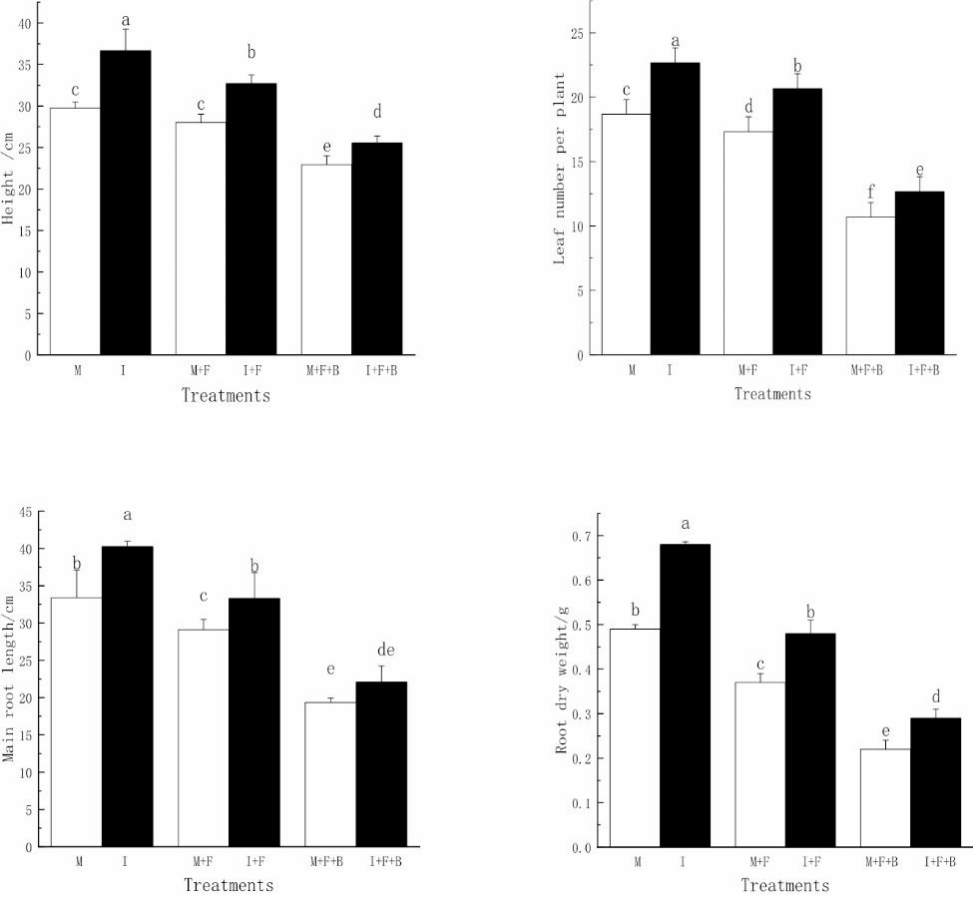
Effects of intercropping on seedling growth of faba bean under benzoic acid stress M: faba bean monocropping; M + F: faba bean monocropping with *FOF* inoculation; M + F + B: faba bean monocropping with *FOF* inoculation and exogenous addition of benzoic acid; I: faba bean-wheat intercropping; I + F: faba bean-wheat intercropping with *FOF* inoculation; I + F + B: faba bean-wheat intercropping with *FOF* inoculation and exogenous addition of benzoic acid

Compared with M treatment, all growth indicators (height, leaf number per plant, main root length and root dry weight) of the faba bean in the M + F treatment were inhibited, which decreased the plant growth by 6.18%~32.43%. After further addition of benzoic acid (M + F + B), The inhibition effect of growth indexes of faba bean was more significant. Compared with monoculture (M,M + F,M + F + B) treatment, intercropping(I,I + F,I + F + B) treatment significantly increased plant height, leaf number per plant, main root length and root dry weight by 11.51%~35% under all treatments (Fig. 1).

### Proteomic analysis of faba bean roots under *FOF* and benzoic acid stress in mono- and intercropping patterns

**Fig. 2:**
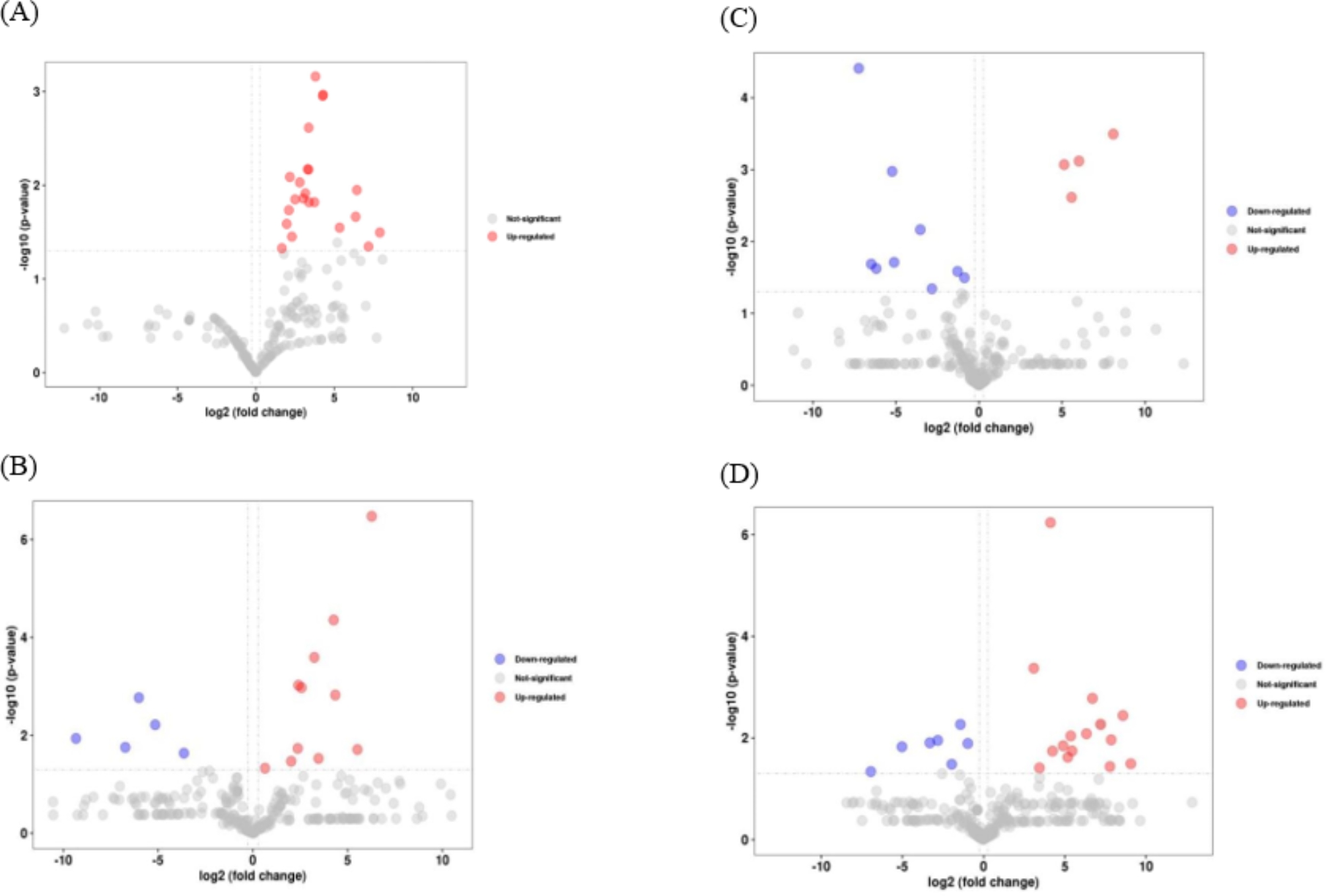
Volcano plot of differentially expressed proteins in the roots of faba bean under *FOF* and benzoic acid stress in monocropping and intercropping patterns. **A**: M+F vs. M, **B**: M+F+B vs. M+F, **C**: I+F+B vs. M+F+B, **D**: I+F vs. M+F. Note. The abscissa is the multiple of the expression difference of the gene / transcript between the two samples, that is, the expression of the treatment sample is divided by the value obtained by the same expression, and the ordinate is the statistical test value of the difference of the change of gene expression, i.e., the p value. The larger the log10 (p value), the more significant the expression difference, and the values of the horizontal and vertical coordinates are logarithmically treated. Each point in the graph represents a specific gene, red dots indicate significantly up-regulated genes, blue dots indicate significantly downregulated genes, and gray dots indicate non-significantly differential genes

Compared with the M treatment, a total of 22 differentially expressed proteins were identified in the M + F treatment, all of which were up-regulated. A total of 16 differentially expressed proteins including 11 up-regulated and 5 down-regulated were identified in the M + F + B treatment compared to the M + F treatment. Compared with the M + F + B treatment, a total of 13 differentially expressed proteins including 4 up-regulated and 9 down-regulated proteins were identified in the I + F + B treatment. Besides, compared with the M + F treatment, a total of 22 differentially expressed proteins, including 15 up-regulated and 7 down-regulated proteins were identified in the I + F treatment (Fig. 2).

### Classification of differentially expressed proteins in faba bean roots via orthologous genes

COG homology analysis of differentially expressed proteins showed that compared with the M treatment, the differential proteins identified in the M + F treatment group were enriched in carbohydrate transport and metabolism, ribosome structure and biogenesis, energy metabolism, amino acid transport and metabolism, nucleotide transport, and metabolism, biosynthesis of secondary metabolites, intracellular transport, translation, and so on. Compared with the M + F treatment, the differential proteins in the M + F + B treatment group were enriched in molecular functions, such as carbohydrate transport and metabolism, energy metabolism, protein conversion, translation, and general function prediction.

**Fig. 3:**
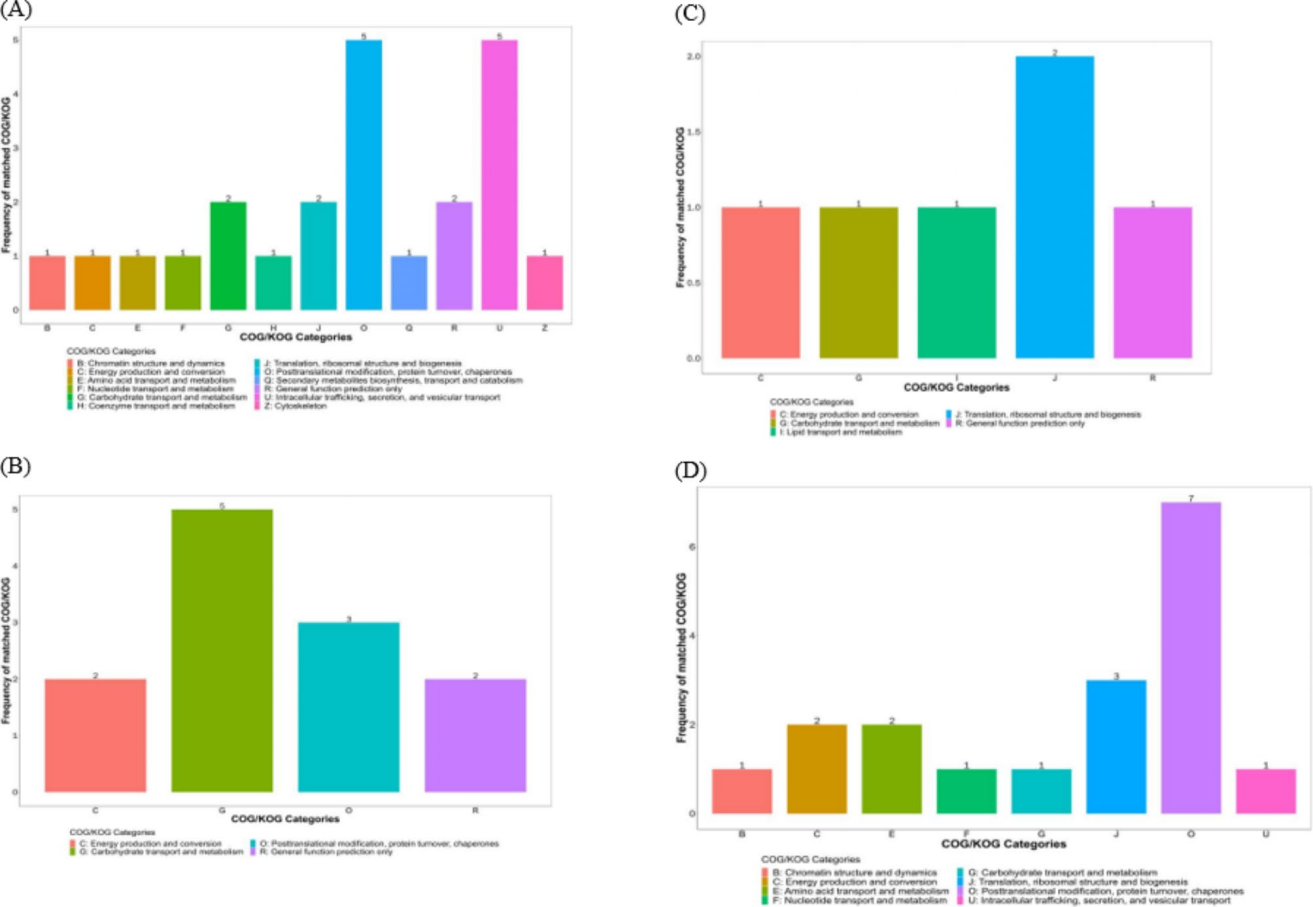
Analysis of homologous genes of differentially expressed proteins in faba bean roots under *FOF* and cinnamic acid stress in mono- and intercropping patterns. **A**: M+F vs. M, **B**: M+F+B vs. M+F , **C**:  I+ F+B vs. M+ F+B, **D**: I+F vs. M+F  . Note. the ordinate represents the functional type of a COG(in capital letters A-Z,see the COG classification statistics);the ordinate indicates the number of genes/transcripts with this function

Compared with the M + F + B treatment, the enriched differential proteins in the I + F + B treatment group were involved in molecular functions, such as carbohydrate transport and metabolism, ribosome structure and biogenesis, energy metabolism, lipid transport and metabolism, translation, and so on. Compared with the M + F treatment, the enriched differential proteins in the I + F treatment were involved in molecular functions, such as carbohydrate transport and metabolism, ribosome structure and biogenesis, energy metabolism, amino acid transport and metabolism, nucleotide transport and metabolism, chromatin structure and kinetics, and so on (Fig. 3).

### Gene ontology (GO) functional enrichment analysis of differentially expressed proteins in faba bean roots

In order to explore which biological processes of differentially expressed proteins participate in faba bean, GO function analysis of differentially expressed proteins was further conducted. The results showed that compared with the M treatment, ten upregulated proteins in the M + F treatment were enriched in the biological process analysis. These enriched proteins were involved in biological processes, such as sucrose metabolism, glucose catabolic process, glycolysis, biosynthesis, hydrogen peroxide catabolic process, carbohydrate metabolism, glycerol catabolic process, and so on. The enriched cellular components for the 11 up-regulated were nucleosome, mitochondria, cytoplasm, nucleus, cell membrane components, cytoplasmic matrix, and so on. The enriched 18 differential proteins were involved in the molecular functions, such as sucrose synthase activity, DNA binding, oxidoreductase activity, metal ion binding, peroxidase activity, chitin binding, hydrolase activity, glycerol kinase activity, and so on (Table 1).

**Table 1:** GO analysis M + F vs. M

		Protein ID	Functional description	Up (↑)/Down (↓)
Biological process(BP)	up-regulated	P31926	Sucrose synthase	↑
		A0A2K3P321	Phosphoglycerate mutase	↑
		Q9SP13	Nucleoside diphosphate kinase	↑
		Q93XK6	Peroxidase	↑
		G7JV88	GTP-binding nuclear protein	↑
		P46298	40 S ribosomal protein S13	↑
		Q2PES6	60 S acidic ribosomal protein P0	↑
		A0A392QW28	Glycoside hydrolase family 18 protein	↑
		A0A1S2YZ86	Lipoxygenase	↑
		A0A072UAH3	3,4-dihydroxy-2-butanone kinase, putative	↑
Cellular component(CC)	up-regulated	Q1RU62	Histone H4	↑
		A0A2Z6MRG8	Expansin-like B1	↑
		A0A2K3P321	Phosphoglycerate mutase	↑
		A0A126TGR5	ATP synthase subunit alpha	↑
		Q93XK6	Peroxidase	↑
		G7JV88	GTP-binding nuclear protein	↑
		P46298	40 S ribosomal protein S13	↑
		Q2PES6	60 S acidic ribosomal protein P0	↑
		A0A2Z6NPJ3	CSD domain-containing protein	↑
		A0A1S2YZ86	Lipoxygenase	↑
		A0A072UAH3	3,4-dihydroxy-2-butanone kinase, putative	↑
Molecular function(MF)	up-regulated	P31926	Sucrose synthase	↑
		Q1RU62	Histone H4	↑
		Q41027	PsHSC71.0	↑
		A0A2Z6MQX4	ADP-ribosylation factor	↑
		A0A126TGR5	ATP synthase subunit alpha	↑
		A0A2Z6NYZ0	Alcohol dehydrogenase 1	↑
		A0A2K3P321	Phosphoglycerate mutase	↑
		Q9SP13	Nucleoside diphosphate kinase	↑
		P51850	Pyruvate decarboxylase 1	↑
		Q66PF9	Monodehydroascorbate reductase I	↑
		Q93XK6	Peroxidase	↑
		G7JV88	GTP-binding nuclear protein	↑
		P46298	40 S ribosomal protein S13	↑
		A0A2Z6N0S0	Disease resistance RPP13-like protein 1	↑
		A0A2K3MIM2	Seed lipoxygenase	↑
		A0A392QW28	Glycoside hydrolase family 18 protein	↑
		A0A2Z6NPJ3	CSD domain-containing protein	↑
		A0A1S2YZ86	Lipoxygenase	↑

Compared with the M + F treatment, a total of 11 enriched proteins including 9 up-regulated proteins and 2 down-regulated proteins in the M + F + B treatment were involved in the biological processes, such as oxidative stress reaction, hydrogen peroxide catabolism, carbohydrate metabolism, D-gluconate catabolism, malic acid metabolism, pyruvate metabolism, flavonoid biosynthesis, and tricarboxylic acid cycle. The proteins related to the assembly process of cell protein complex were up-regulated and the proteins related to carbohydrate metabolism and glycerol catabolism were down-regulated. A total of 11 differential proteins including 8 up-regulated proteins and 3 down-regulated proteins were enriched in the cell component analysis. The cellular components that were enriched for the upregulated differential proteins included the cell membrane component, chloroplast outer membrane of the chloroplast, mitochondria, cytoplasmic matrix, endoplasmic reticulum membrane protein complex, and for the down-regulated proteins included ATP synthase, membrane, and cytoplasmic matrix. The molecular function analysis of the differential proteins showed that the ten enriched up-regulated proteins were involved in molecular functions, such as malate dehydrogenase, metal ion binding, chalketone isomerase activity, ketoglutarate dehydrogenase, peroxidase activity and heme binding, and five enriched down-regulated proteins were involved molecular functions, such as ATP binding, oxidoreductase activity, chitin binding, hydrolase activity, nucleic acid binding, glycerol kinase activity, and so on (Table 2).

**Table 2:** GO analysis M + F + B vs. M + F

		Protein ID	Functional description	Up(↑)/Down (↓)
Biological process(BP)	up-regulated	A0A072TX81	6-phosphogluconate dehydrogenase, decarboxylating	↑
		G7JEN5	Transketolase	↑
		A0A2K3M2W9	Peroxidase	↑
		G7JCF5	Protein TOC75	↑
		G7L7H0	Malic enzyme	↑
		A0A173H220	Chalcone-flavonone isomerase family protein	↑
		G7KVS0	Oxoglutarate dehydrogenase (succinyl-transferring)	↑
		Q18PR1	Peroxidase	↑
		A0A3Q7YH50	beta-xylosidase/alpha-L-arabinofuranosidase 1 isoform X2	↑
	down-regulated	A0A392QW28	Glycoside hydrolase family 18 protein	↓
		A0A072UAH3	3,4-dihydroxy-2-butanone kinase, putative	↓
Cellular component(CC)	up-regulated	A0A072TX81	6-phosphogluconate dehydrogenase, decarboxylating	↑
		G7L7H0	Malic enzyme	↑
		A0A2K3L0E9	Er membrane protein complex subunit 8/9	↑
		G7JCF5	Protein TOC75	↑
		G7KVS0	Oxoglutarate dehydrogenase (succinyl-transferring)	↑
		G7JEN5	Transketolase	↑
		Q18PR1	Peroxidase	↑
		A0A3Q7YH50	beta-xylosidase/alpha-L-arabinofuranosidase 1 isoform X2	↑
	down-regulated	A0A126TGR5	ATP synthase subunit alpha	↓
		A0A2Z6NPJ3	CSD domain-containing protein	↓
		A0A072UAH3	3,4-dihydroxy-2-butanone kinase, putative	↓
Molecular function(MF)	up-regulated	Q41027	PsHSC71.0	↑
		A0A072TX81	6-phosphogluconate dehydrogenase, decarboxylating	↑
		G7JEN5	Transketolase	↑
		A0A2K3M2W9	Peroxidase	↑
		G7JCF5	Protein TOC75	↑
		G7L7H0	Malic enzyme	↑
		A0A173H220	Chalcone-flavonone isomerase family protein	↑
		G7KVS0	Oxoglutarate dehydrogenase (succinyl-transferring)	↑
		Q18PR1	Peroxidase	↑
		A0A3Q7YH50	beta-xylosidase/alpha-L-arabinofuranosidase 1 isoform X2	↑
	down-regulated	A0A126TGR5	ATP synthase subunit alpha	↓
		A0A2Z6LMQ0	Pyr_redox_2 domain-containing protein	↓
		A0A392QW28	Glycoside hydrolase family 18 protein	↓
		A0A2Z6NPJ3	CSD domain-containing protein	↓
		A0A072UAH3	3,4-dihydroxy-2-butanone kinase, putative	↓

Compared with the M + F + B treatment, in the I + F + B treatment 2 up-regulated proteins that were enriched in the biological process analysis were involved in carbohydrate metabolism, hydrogen peroxide catabolism, and reaction to oxidative stress, and 7 enriched down-regulated proteins were involved in cellular redox homeostasis, oxidative stress response, hydrogen peroxide catabolism, carbohydrate metabolism, glycolysis, fatty acid biosynthesis, translation and so on. In the cellular component analysis of the 6 differentially expressed proteins, the outer membrane of mitochondria was enriched for 1 up-regulated protein, and the endoplasmic reticulum membrane protein complex, extracellular region, and cell membrane components were enriched for down-regulated proteins. In the molecular function analysis of 12 proteins, enriched up-regulated proteins were involved in GTP enzyme activity, GTP binding, chitin binding, hydrolase activity, metal ion binding, peroxidase activity, and so on and downregulated proteins were involved in magnesium ion binding, dihydroacyl dehydrogenase activity, rRNA binding, peroxidase activity, heme binding, metal ion binding, and so on (Table 3).

**Table 3:** GO analysis I + F + B vsM + F + B

		Protein ID	Functional description	Up(↑)/Down (↓)
Biological process(BP)	up-regulated	A0A392QW28	Glycoside hydrolase family 18 protein	↑
		A0A2K3M8I6	Peroxidase	↑
	down-regulated	A0A072U338	Phosphopyruvate hydratase	↓
		A0A2K3MC91	Dihydrolipoyl dehydrogenase mitochondrial-like	↓
		A0A1S2YRH3	40 S ribosomal protein S9-2-like	↓
		A0A2K3M2W9	Peroxidase	↓
		Q18PR1	Peroxidase	↓
		A0A3Q7YH50	beta-xylosidase/alpha-L-arabinofuranosidase 1 isoform X2	↓
		A0A1S2Y945	Acetyl-CoA carboxylase	↓
Cellular component(CC)	up-regulated	A0A3Q7 × 702	mitochondrial outer membrane protein porin 4-like	↑
	down-regulated	A0A072U338	Phosphopyruvate hydratase	↓
		A0A1S2YRH3	40 S ribosomal protein S9-2-like	↓
		A0A2K3L0E9	Er membrane protein complex subunit 8/9	↓
		Q18PR1	Peroxidase	↓
		A0A3Q7YH50	beta-xylosidase/alpha-L-arabinofuranosidase 1 isoform X2	↓
Molecular function(MF)	up-regulated	A0A2Z6N628	Uncharacterized protein	↑
		A0A392QW28	Glycoside hydrolase family 18 protein	↑
		A0A3Q7 × 702	mitochondrial outer membrane protein porin 4-like	↑
		A0A2K3M8I6	Peroxidase	↑
	down-regulated	A0A072U338	Phosphopyruvate hydratase	↓
		A0A2K3MC91	Dihydrolipoyl dehydrogenase mitochondrial-like	↓
		A0A1S2YRH3	40 S ribosomal protein S9-2-like	↓
		A0A2K3M2W9	Peroxidase	↓
		A0A2Z6NJR2	Uncharacterized protein	↓
		Q18PR1	Peroxidase	↓
		A0A3Q7YH50	beta-xylosidase/alpha-L-arabinofuranosidase 1 isoform X2	↓
		A0A1S2Y945	Acetyl-CoA carboxylase	↓

Compared with the M + F treatment, biological process analysis of ten differential proteins in the I + F treatment including 8 up-regulated and 2 down-regulated was also performed. The enriched up-regulated proteins were involved in the biological processes, such as carbon fixation, tricarboxylic acid cycle process, biosynthesis process, cellular amino acid metabolism process, proteasome protein catabolism process, proteasome ubiquitin-independent protein catabolism process, ubiquitin-dependent protein catabolism process, carbohydrate metabolism process, and so on and enriched down-regulated proteins were involved in oxidized lipid biosynthesis and ribosome synthesis.

A total of thirteen differential proteins, including 9 up-regulated proteins and 4 down-regulated proteins were enriched in the cellular component analysis. Out of these proteins, cytoplasmic components, nuclear and proteasome core complexes, extracellular regions, ribosomes, mitochondria, and membrane components were enriched for the up-regulated proteins and nucleosomes, ribosomes, membrane components, and cytoplasm were enriched for the down-regulated proteins. The molecular function analysis of a total of sixteen differential proteins including 11 up-regulated proteins and 5 down-regulated proteins showed that enriched up-regulated proteins were involved in functions, such as pyruvate carboxylase activity, endopeptidase activity, GTPase activity, calcium binding, β furanosidase activity, the structural composition of ribosomes, the activity of kinase and the activity of cytochrome c oxidase, whereas enriched downregulated proteins were involved in DNA binding, oxidoreductase activity, metal ion binding, nucleic acid binding, heme binding, and iron ion binding (Table 4).

**Table 4:** GO analysis I + F vs. M + F

		Protein ID	Functional description	Up(↑)/Down (↓)
Biological process(BP)	up-regulated	O82723	Phosphoenolpyruvate carboxylase	↑
		A0A1S2Y9Y3	Aspartate aminotransferase	↑
		A0A1S2XI32	Proteasome subunit alpha type	↑
		I3SSX1	Proteasome subunit alpha type	↑
		Q43089	Beta-fructofuranosidase, cell wall isozyme	↑
		A0A2K3K510	60 S ribosomal protein L27	↑
		A0A2K3LR80	Cytochrome c oxidase subunit 3	↑
		A0A392LZ79	Proteasome subunit alpha type	↑
	down-regulated	Q2PES6	60 S acidic ribosomal protein P0	↓
		A0A1S2YZ86	Lipoxygenase	↓
Cellular component(CC)	up-regulated	O82723	Phosphoenolpyruvate carboxylase	↑
		A0A1S2XI32	Proteasome subunit alpha type	↑
		Q6ISX8	Kunitz type trypsin inhibitor	↑
		I3SSX1	Proteasome subunit alpha type	↑
		A0A2K3LR80	Cytochrome c oxidase subunit 3	↑
		A0A392LZ79	Proteasome subunit alpha type	↑
		A0A392PS94	Succinate dehydrogenase 6	↑
		A0A2K3PPS2	Lipoxygenase y domain-containing protein 1-like	↑
	down-regulated	Q1RU62	Histone H4	↓
		Q2PES6	60 S acidic ribosomal protein P0	↓
		A0A2Z6NPJ3	CSD domain-containing protein	↓
		A0A1S2YZ86	Lipoxygenase	↓
Molecular function(MF)	up-regulated	O82723	Phosphoenolpyruvate carboxylase	↑
		A0A1S2Y9Y3	Aspartate aminotransferase	↑
		Q6ISX8	Kunitz type trypsin inhibitor	↑
		I3SSX1	Proteasome subunit alpha type	↑
		A0A2Z6N628	Uncharacterized protein	↑
		A0A2K3LR80	Cytochrome c oxidase subunit 3	↑
		A0A1S2YFJ4	Annexin	↑
		G7KNI3	Cytosolic aldehyde dehydrogenase	↑
		Q43089	Beta-fructofuranosidase, cell wall isozyme	↑
		A0A2K3K510	60 S ribosomal protein L27	↑
		A0A392N2Q9	Putative serine/threonine kinase plant-type protein	↑
	down-regulated	Q1RU62	Histone H4	↓
		A0A2K3MIM2	Seed lipoxygenase	↓
		A0A2Z6NPJ3	CSD domain-containing protein	↓
		A0A1S2YZ86	Lipoxygenase	↓

A total of thirteen differential proteins, including 9 up-regulated proteins and 4 down-regulated proteins were enriched in the cellular component analysis. Out of these proteins, cytoplasmic components, nuclear and proteasome core complexes, extracellular regions, ribosomes, mitochondria, and membrane components were enriched for the up-regulated proteins and nucleosomes, ribosomes, membrane components, and cytoplasm were enriched for the down-regulated proteins. The molecular function analysis of a total of sixteen differential proteins including 11 up-regulated proteins and 5 down-regulated proteins showed that enriched up-regulated proteins were involved in functions, such as pyruvate carboxylase activity, endopeptidase activity, GTPase activity, calcium binding,β furanosidase activity, the structural composition of ribosomes, the activity of kinase and the activity of cytochrome c oxidase, whereas enriched downregulated proteins were involved in DNA binding, oxidoreductase activity, metal ion binding, nucleic acid binding, heme binding, and iron ion binding (Table 4).

### Kyoto encyclopedia of genes and genomes (KEGG) functional enrichment analysis of differentially expressed proteins in faba bean roots

KEGG enrichment analysis of the differential proteins in the M + F treatment showed enrichment of four KEGG pathways, i.e., oxidative phosphorylation, metabolic pathway, ribosomal synthesis, and RNA transport pathway. Compared with the M + F treatment, the KEGG pathway analysis of the differentially expressed proteins in the M + F + B treatment showed the enrichment of ten pathways, including metabolic pathways, carbon metabolism, amino acid biosynthesis, and pyruvate metabolism.

**Fig. 4:**
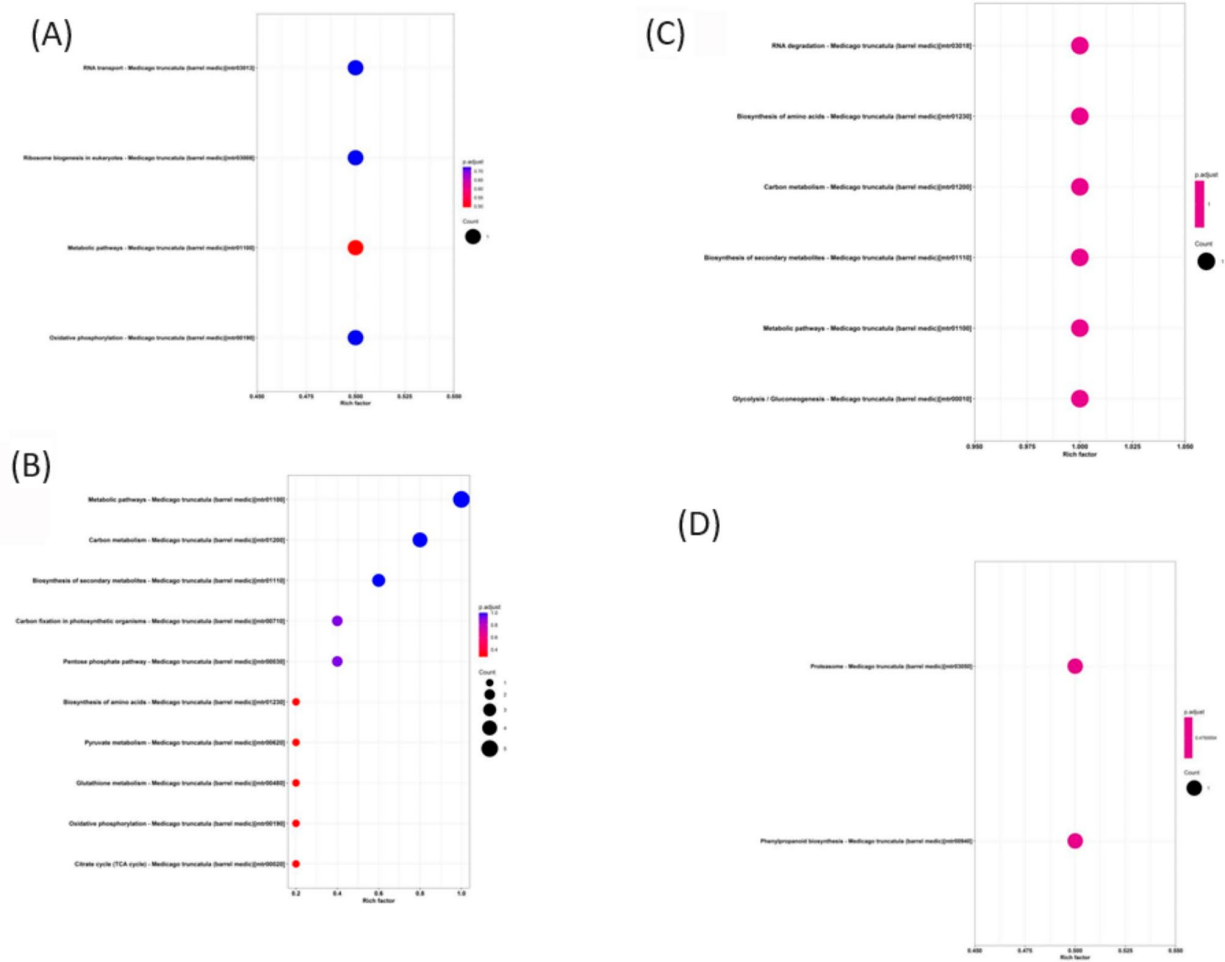
Enrichment of differential protein pathways in faba bean roots under *FOF* and benzoic acid stress. **A**: M + F vs.M **B**:M + F + B vs.M + F **C**:I + F + B vs.M + F + B **D**:I + F vs.M + F. The horizontal axis Rich factor represents the number of different genes corresponding to the pathway, and the vertical axis represents the enriched description information of the pathway. Count represents the number of enriched genes. p.adjust specifies the number of pathways displayed. Different colors represent different adjusted p-values. From large to small, the degree of enrichment becomes more and more significant

Compared with the M + F + B treatment, the KEGG pathway analysis of the differentially expressed proteins in the I + F + B treatment showed enrichment of metabolic pathways, biosynthesis of secondary metabolites, biosynthesis of amino acids, and RNA degradation. Compared with the M + F treatment, the KEGG pathway analysis of the differentially expressed proteins in the I + F treatment showed enrichment of two pathways, i.e., proteasome and phenylpropane biosynthesis pathways (Fig. 4).

### Interaction of the differentially expressed proteins in faba bean roots

**Fig. 5:**
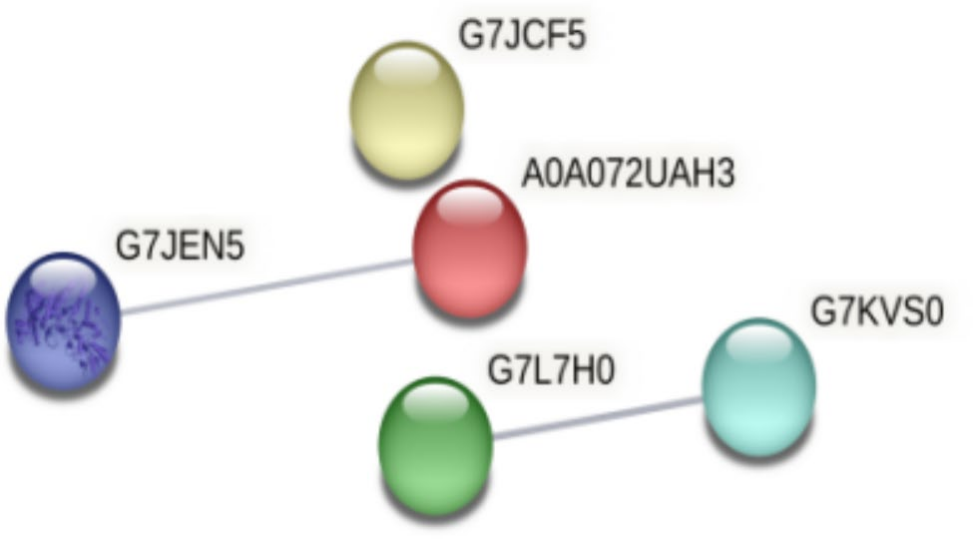
Interaction among differentially expressed proteins in faba bean roots

Interaction analysis of differentially expressed proteins, in which interacting proteins were identified in the M + F + B treatment compared to the M + F treatment, showed that there were a total of three interacting nodes of differentially expressed proteins in the M + F + B treatment, i.e., (a) G7L7H0 and G7KVS0, (b) G7JEN5 and A0A072UAH3, and (c) G7JEN5 and G7KVS0 compared to the M + F treatment group. These interacting proteins were malic enzyme, transketolase, ketoglutarate dehydrogenase, and 3,4-dihydroxy-2-butanone kinase with functions mainly in molecular functions and maintenance of intracellular redox homeostasis (Fig. 5).

## Discussion

With the rise of continuous cropping years of faba bean, the accumulation of autotoxic substances in soil has increased the incidence of faba bean fusarium wilt. Faba bean and wheat are typically intercropped in Southwest China and it has shown increased crop yield and controlled plant diseases. Previous studies have shown that the content of benzoic acid in the rhizosphere soil of mono-cropped faba beans was relatively high [22]. Thus, in this study, we hypothesized that benzoic acid is an important autotoxic substance promoting the development of faba bean fusarium wilt. The role of benzoic acid in promoting the development of faba bean fusarium wilt and the potential mechanism of faba bean wheat intercropping in controlling the development of faba bean fusarium wilt was also explored in this study.

### The growth of faba bean seedlings was inhibited under *FOF* stress due to severe cell oxidation

The occurrence of plant soil-borne diseases is usually caused by the fungal infection of plant roots and stems [23]. Thus, we investigated the changes of protein in faba bean root under pathogen stress alone. We found that compared with the control treatment (M), the function of overexpressed proteins in the *FOF* treatment (M + F), included redox, energy metabolism, hydrogen peroxide catabolism process, and response to oxidative stress. This indicated that after inoculation with *FOF*, the fine cells of faba bean were seriously damaged by oxidation, and the increased degree of cell peroxidation, increasing the peroxidation pressure on the cell membrane and organelles, and in turn destroying the stability of cell membrane. The outcomes of the current study are in line with the previous study [24, 25]. Lu Y et al. [24] reported that differential proteins in *FOF*-infected banana plants were mainly related to metabolism, redox, immunity, and defense. These proteins play a crucial role in imparting resistance to banana plants against fusarium wilt. Silva et al. [25] reported that the differential proteins in the root of tomato played an important role in metabolism, redox balance, energy, and defense after inoculation with *FOF*. In our study, the up-regulated expression of these proteins is the stress response of the faba bean to the stress of *FOF*, which improves the anti-oxidation ability of the faba bean and maintains the balance of intracellular active oxygen metabolism.

### Dual stress of *FOF* and benzoic acid led to more serious oxidative damage of faba bean plants and severe toxic effects

Previous studies have shown that in addition to a large number of pathogenic bacteria in continuous cropping soil, plant roots also secrete a plethora of phenolic acids, such as ferulic acid, cinnamic acid, salicylic acid, and so on [26]. In actual production, faba bean plants are not only subjected to individual stress from pathogens, but also to dual stress from pathogens and autotoxicity substances. Therefore, we investigated the growth and development, and the changes in root proteins of faba beans under the dual stress of FOF and benzoic acid. We found that compared with the *FOF* treatment (M + F), after further exposure to benzoic acid stress (M + F + B), the growth indicators of faba bean seedlings were significantly reduced. This finding is in line with the results of the current study. Zhou et al. [5] have shown that vanillic acid secreted by cucumber roots promotes the growth of pathogenic bacteria, significantly inhibiting the growth and development of its seedlings, and increasing the risk of cucumber infection with fusarium wilt. After treatment with ferulic acid, *Rehmannia glutinosa* significantly increased its ability to produce mycotoxins, inhibiting its growth and increasing the incidence rate of Fusarium wilt [27]. This indicated that benzoic acid had a severe toxic effect on faba bean seedlings, significantly inhibiting their plant growth.

In this study, compared with the *Fusarium oxysporum f. sp. fabae* treatment (M + F), under the double stress of *Fusarium oxysporum f. sp. fabae* and benzoic acid (M + F + B), the functions mainly focus on the up-regulation of the related proteins of intracellular redox balance, defense, cell wall function, and maintenance of cell integrity. This indicated that with further benzoic acid stress, the oxidative damage of plant cells is aggravated, the normal physiological and metabolic pathways in faba beans are disrupted, then it initiate its own defense response to exerting the tissue resistance of the cell wall to protect it from damage, exerting the redox level in cells normal,and maintaining the plant growth. Previous studies have reported that the differential proteins in rice roots under ferulic acid stress were mainly enriched in redox, metabolism, protein hydrolysis, and other processes. This in turn induces the synthesis of numerous free radical scavenging enzymes and antioxidant enzymes to regulate stress [28]. Under phthalic acid stress, the root differential proteins of roots of Taraxacum mongolicum seedlings mainly participate in the defense, translation, redox balance, and other processes, and their antioxidant enzyme activity, cell membrane structure, and function are damaged [29]. This is similar to our findings. In this study, we identified antioxidant enzymes, such as SOD, POD, CAT, and oxygen free radicals’ scavengers. These enzymes maintain active oxygen metabolism in plants and play an important role in scavenging active oxygen in plants. The differential expression of SOD, POD and CAT enzymes indicated severe oxidative damage in the *FOF*-infected and benzoic acid-poisoned faba bean. After the expression of SOD, POD, and CAT, the oxidation level returned to normal, relieved the oxidative stress of faba bean root cells, and increased the resistance of faba bean.

### Intercropping can help faba bean resist cell damage caused by peroxidation and promote the synthesis of ethylene and jasmonic acid

Numerous studies have shown that intercropping can effectively control the occurrence of plant diseases and promote plant growth. Therefore, we investigated the growth and development, and the changes in root proteins of faba beans under intercropping conditions. In each treatment, compared with monocropping, the intercropping of faba bean and wheat significantly increased the plant height, number of leaves per plant, main root length, and root dry weight of faba bean seedlings, and reduced the inhibition due to *FOF* and benzoic acid on the seedling growth. Chuan et al. [30] have reported that intercropping peanut with *Atractylodes lancea* significantly improves the growth of peanut seedlings and reduce the incidence rate of peanut root rot. Zhu et al. [31]have shown that the number of ears, 100-grain weight, and plant height of maize significantly increased in the intercropping of maize and pea. These findings are in line with the outcomes of the current study. In the intercropping system, the growth indexes of faba bean seedlings were higher than those of single cropping, the reason could be the intercropping system of faba bean and wheat. The interaction between *FOF* and benzoic acid in the roots alleviates the damage to the root of the faba bean and the stress on the growth of the aboveground part, promoting its growth.

Compared with the M + F + B treatment, the differential proteins in the I + F + B treatment were involved in energy metabolism, cell integrity, and oxidation-reduction balance. This indicates that intercropping could help the faba bean in resisting the cellular damage caused due to peroxidation, mitigating the stress caused due to *FOF* and benzoic acid on faba bean, and controlling the occurrence of faba bean fusarium wilt. In addition, compared with monocropping, the intercropping of faba bean and wheat also improved the resistance of faba bean by improving the biosynthesis of secondary metabolites. We also found that KEGG pathway enrichment analysis of upregulated proteins in the I + F treatment showed enrichment of multiple pathways, such as energy, photosynthetic carbon fixation, biosynthesis of secondary metabolites, jasmonate synthesis, and ethylene synthesis. This indicated that stress was caused due to *FOF*-activated jasmonate, ethylene, and other resistance pathways. Faba bean increased its resistance to fusarium wilt by synthesizing jasmonic acid and ethylene, maintaining the growth of faba bean plants. This is consistent with current research studys. Yao et al. [32] have shown that the intercropping of eucalyptus and *Dalbergia odorifera* increased the biosynthesis of jasmonic acid in its roots which increased the plant growth. Hu et al. [33] have shown that the secondary metabolites secreted by maize increase the jasmonic acid synthesis in offspring plants, enhancing the plant defense in maize. This is in line with the outcomes of the current study. These results indicated that the intercropping of faba bean and wheat helped controlled the occurrence of fusarium wilt by enhancing the antioxidant stress capacity of faba bean roots. It also significantly promoted the synthesis of proteins related to the production of secondary metabolites in the root of the faba bean and enhanced the disease resistance of the faba bean root. This could be a crucial disease control mechanism of faba bean wheat intercropping.

## Conclusion

This study showed that after inoculation with *FOF*, the growth of faba beans was significantly inhibited, and faba beans initiated a defense response to *FOF* stress by up-regulating proteins involved in functions, such as redox and cell wall function. After further addition of benzoic acid, the stability of the cell membrane and defense system of the faba bean root system was disrupted. Faba bean responded to the dual stress of *FOF* and benzoic acid by up-regulating proteins related to defense, redox, and energy. However, the intercropping of faba bean and wheat promoted the synthesis of ethylene and jasmonic acid, improved the resistance of faba bean plants, and effectively alleviated the stress of *FOF* and benzoic acid. This inhibited the occurrence of faba bean fusarium wilt by up-regulating the biosynthesis of secondary metabolites, carbon fixation of photosynthesis, and other related proteins. This study analyzed altered protein expression during the interaction of faba bean-*Fusarium oxysporum f. sp. fabae*-autotoxins. This study also explored the mechanism of controlling the occurrence of faba bean fusarium wilt through intercropping between faba bean and wheat, providing scientific and reasonable theoretical support for intercropping to control the disease and determination of jasmonic acid and ethylene content.

## Materials and methods

### Experimental design of hydroponic potted plants

#### Test materials

The faba bean (*Vicia faba* L.) and wheat (*Triticum aestivum* L.) varieties used in the experiment were “89–147” and “Yunmai 56”. Faba bean and wheat seeds were purchased from the Food Crops Research Institute of Yunnan Academy of Agricultural Sciences. The phenolic acid standard was detected using high-performance liquid chromatography (HPLC) at the level of chromatographic purity. Phenolic acid was purchased from Sigma Corporation, USA. The benzoic acid used in this study was purchased from Sinopharm Shanghai Co.Ltd. The specific type of *Fusarium oxysporumf. sp.fabae* (*FOF*) used in this experiment was screened and preserved from the continuous cropping soil of faba beans. The fungus was transferred to potato dextrose agar (PDA) media, incubated at 28 ℃ for 7 days, and stored at 4 ℃.

#### Experimental design

The experiment was conducted in a glass greenhouse at Yunnan Agricultural University from October 2019 to February 2020. Faba beans and wheat plants were grown in M (6 faba bean plants per pot) or I (3 faba bean plants and 9 wheat plants per pot) pattern. Faba beans and wheat were grown under (a) no stress (M, F), (b) *FOF* stress alone (M + F, I + F), and (c) *FOF* and benzoic acid double stress (M + F + B, I + F + B) treatments at 26 ℃/22 ℃ light for 14 h per day. Each treatment was replicated three times. Faba bean seedlings were transplanted into pots containing 2 L of the nutrient solution once they reached 4–6 true leaves. Later, benzoic acid was added exogenously and *FOF* was inoculated after 2 d of benzoic acid treatment. The concentration of benzoic acid was 100 mg·L^− 1^. A total of 25 mL of *Fusarium* acnes spore suspension at a concentration of 1 × 10^6^ CFU/mL was inoculated into the nutrient solution [34]. The nutrient solution was changed every 5 d. Benzoic acid was re-added and *FOF* was inoculated when the nutrient solution was changed, and the plants were aerated with an aeration pump throughout the hydroponic process.

The Hoagland Nutrient Solution formula contained 0.75 mmol/L of K_2_SO_4_, 0.65 mmol/L of MgSO_4_, 0.1 mmol/L of KCl 0.1, 0.25 mmol/L of KH_2_PO_4_, 0.001 mmol/L of H_3_BO_4_, 0.001 mmol/L of MnSO_4_, 0.0001 mmol/L of CuSO_4_, 0.001 mmol/L of ZnSO_4_, 0.000005 mmol/L of (NH_4_)_6_Mo_7_O_24_, and 0.2 mmol/L of Fe-EDTA.

#### Measurement of growth indexes of the faba bean seedlings

Faba bean samplings were grown after 30 d of benzoic acid treatment and three plants were randomly selected from each replicate to measure and record their plant height, the number of leaves per plant, and main root length. Later, the sampled plants were rinsed with distilled water and then the above-ground and below-ground parts of the plants were packed inside paper bags that were tagged with numbers. These samples were dried using a blast oven at 105 °C for 30 min and later at 65 °C till the samples attained a constant weight and then the above-ground and below-ground parts of the plants were weighed and recorded.

### Proteomic analysis of faba bean root system

The sampling time in the faba bean root system is the same as 1.2. Three faba bean strains were randomly selected for each replicate. Fresh root samples of the faba bean plants were cut with sterilized scissors. Around 5 g of the fresh root samples were stored in the frozen storage tube and then stored in a liquid nitrogen tank for later use. The proteomic analysis of the faba bean roots was performed by Shanghai Baiqu Biomedical Technology Co. Ltd. (website: www.biotree.cn). The experimental procedures are as follows.

#### Protein extraction

An appropriate amount of sample was taken into a new EP tube. An appropriate amount of phenol extract and protease inhibitor was added to this sample and the mixture was ground for 5 min at a low temperature. An equal volume of tris (pH 8.0) was added to balance the phenol-saturated solution and incubated for 30 min at 4 °C with continuous shaking every 5 min. The resulting solution was centrifuged at 7200 g at 4 ℃ for 15 min and the upper layer was separated and collected. Later, a precooled 0.1 M ammonium acetate-methanol solution was added and precipitated at -20 ℃ overnight. This was followed by centrifugation at 12,000 RPM at 4 ℃ for 10 min, precipitation, and the addition of pre-cooled methanol. The resulting solution was again centrifuged at 12,000 g at 4 ℃ for 10 min, the supernatant was precipitated and the process was repeated. Methanol was replaced with acetone and the process was repeated twice. This solution was centrifuged at 4 °C for 10 min, the precipitate was separated, and dried for 1 min. The dried powder was dissolved in the sample pyrolysis solution, blown, mixed, and sonicated for 5 min. The resulting solution was centrifuged at 12,000 g at 4 °C for 10 min, the supernatant was collected into a new EP tube.

#### Proteolytic hydrolysis

A total of 1 µL of 1 µg/µL trypsin was added to the EP tube and incubated in the water bath at 37 ℃ for 12 h. The resulting solution was centrifuged at 5000 g and the supernatant was separated in the EP tube. The peptide solution was dried into a powder and frozen using the freeze dryer.

#### Peptide desalt

1. The dried peptide segment was dissolved in 100 µL of 0.1% TFA and 0.5% of acetonitrile solution. The desalting column was activated using100 µL of 0.1% TFA and 60% acetonitrile. To balance the desalting column, 200 µL of 0.1% TFA, and 1% acetonitrile solution were added to the desalting column. The trypsin-digested sample was added to the desalting column to capture the peptide segment, and the outflow salt and other small molecules were discarded. The desalting column was washed using 100 µL of 0.1% TFA, and 0.5% of acetonitrile solution and residual salt was removed. 150 µL of 0.1% TFA and 60% of acetonitrile solution were used to make liquid flow slowly through the desalting column and elute the peptide segment. The elute was collected in a new EP tube.
2. Freezing and drying the elute to remove acetonitrile.

#### Mass spectrometry detection

Around ~ 1 µg of the total peptide of each sample was analyzed, separated using the nano UPLC liquid system EASYnLC1200 and coupled using a mass spectrometer (QExactive HFX) equipped with a nanoliter ion source for data acquisition. Chromatographic separation was performed on a 100 μm ID × 15 cm reversed-phase column (Reprosil Pur 120 C18AQ, 1.9 μm, Dr. Maisch). Acetonitrile-water-formic acid was used as the mobile phase. Mobile phase A was 0.1% formic acid − 98% water (2% acetonitrile) and mobile phase B was 0.1% formic acid − 80% acetonitrile (20% water). After equilibrating the column with 100% of phase A, the sample was directly injected into the column using the autosampler and then separated using the column gradient at a flow rate of 300 nL/min and a gradient duration of 120 min. The proportion of mobile phase B: 2–5% for 2 min, 5–22% for 88 min, 22–45% for 26 min, 45–95% for 2 min, and 95% for 2 min.

The mass spectrometry analysis was performed in data-dependent acquisition (DDA) mode with a total analysis time of 120 min in positive ion detection mode. A full scan was performed in the range of 350–1600 m/z with a resolution of 120k (200 m/z), an AGC of 3E6, and a maximum ion injection time (max IT) of 50 ms. The 20 ions (top 20) with the highest intensity in the primary scanning were screened using the quadrupole and cleaved using HCD for fragment ion scanning. The quadrupole isolation window was 1.2 m/z, the standardized collision energy (NCE) was 27%, the AGC was 1E5, and the max IT was 110 ms. The resolution of the secondary scan was set to 15 k. The dynamic exclusion time was set to 45 s according to the peak width, ions with a single charge, and > 6 valence were not subjected to the secondary scan 2.

### Data statistics and analysis

The data were analyzed using Excel 2013. All data were tested for normality. ANOVA was analyzed using the SPSS version 23.0. Independent samples T-test was used to analyze the same phenolic acid for single and intercropping treatments, and one-way ANOVA was used to analyze different treatments for the same growth index. All differences were considered significant when P < 0.05. Statistical methods (t-test) are used to screen differentially expressed proteins, and the screening criteria for differentially expressed proteins were P-VALUE < 0.05, and FOLD CHANGE < 0.83 or FOLD CHANGE > 1.2. Functional annotation of differentially expressed proteins from UNIPROT database (https://www.uniprot.org/).

## Data Availability

All data generated or analysed during this study are included in this published article.

## **Abbreviations**

FOF: *Fusarium oxysporum f. sp. fabae*
